# Interaction between dietary digestible tryptophan and soy oligosaccharides in broiler chickens: effects on caecal skatole level and microflora

**DOI:** 10.5713/ab.22.0060

**Published:** 2022-09-02

**Authors:** Jing Chen, Hansong Jing, Haiying Liu, Xin Zhu, Guiqin Yang

**Affiliations:** 1 College of Animal Science and Veterinary Medicine, Shenyang Agricultural University, Shenyang, Liaoning 110866, China

**Keywords:** Aromatic Amino Acid, Growth Performance, Non-digestible Carbohydrates, Odour-causing Compounds, Volatile Fatty Acids

## Abstract

**Objective:**

This study was conducted to evaluate the interactive effects of dietary digestible tryptophan (dTry) and soy oligosaccharides (SO) on growth performance, caecal skatole level, and microflora of broiler chickens aged from 14 to 42 days.

**Methods:**

Three hundred and sixty broiler chicks were allocated equally to 36 cages at 14-day-of-age according to body weight and gender. Using a 3×2 factorial arrangement, 3 dietary dTry levels (0.18%, 0.23%, and 0.28%) supplemented with 0 or 3.5 g/kg of SO were used to create 6 diets (treatments). Each diet was fed to six replicates of 10 birds (60 birds/treatment), growth performance was measured. Caecal content samples were collected at 42 days of age.

**Results:**

Results showed that significantly different dTry level×SO interactions were found for average daily gain (ADG), caecal levels of indole, propionic acid, and butyric acid, and microbial Shannon index (p<0.05). Birds fed diet containing 0.23% dTry level with SO supplementation had higher ADG and lower feed/gain ratio than those fed the other diets (p<0.05). Broilers fed diets containing 0.28% dTry increased their caecal levels of indole and skatole compared with those containing 0.18% or 0.23% dTry (p<0.01), regardless of SO addition. SO supplementation to diets decreased the caecal skatole level by 16.17% (p<0.05), and increased the relative frequency of *Clostridium IV* (p<0.05), regardless of dietary dTry level.

**Conclusion:**

These results indicated that diets containing 0.23% dTry with SO supplementation positively promoted ADG, and decreased caecal skatole levels of broiler chickens. The dietary dTry level, SO affected the caecal skatole level, however, there was no interaction between them.

## INTRODUCTION

Odour emissions have been identified as a potential threat to the sustainable development of the animal industry [1]. Emissions from farms raising broilers contain a large number of odourants [2], which include hydrocarbons, aldehydes, ketones, sulphur compounds and heterocycles [3]. Skatole (3-methylindole), a compound formed by the bacterial degradation of L-tryptophan [4], can be detected by the human olfactory system at a threshold of 3 μg/m^3^ [5]. Skatole is the main contributor to malodorous chicken excreta [6–8], it not only reduces the production performance and product quality of animals but also endangers the health of animals and surrounding residents [9]. Therefore, exploring caecal-skatole reduction strategies is vital to reduce potential pollution of animal husbandry related activities.

Tryptophan is an essential amino acid for humans and animals. Studies have shown that the appropriate digestible tryptophan (dTry) levels varied from 0.11% to 0.23% in typical maize and soybean-meal-based diets for broilers [10,11]. Khattak and Helmbrecht [4] indicated that there was a significant positive linear correlation between skatole and dietary tryptophan level in laying hens. A high level of dietary amino acids may escape the host digestion [12]. A lower crude protein diet was effective in reducing the emissions of phenol, indole, skatole, and others from broiler chicken excreta [1]. Our previous study indicated significant positive correlations between skatole level and intestinal microflora diversity, richness, and total bacterial count [13].

Soy oligosaccharides (SO) is a group of non-digestible carbohydrates that are extracted from soybean seeds or other legumes, primarily consisting of sucrose, stachyose, and raffinose. As non-digestible structures, they provide carbohydrates for the distal intestine, thus suppressing putrefaction [12]. It is known that the inclusion of 3.5 to 5.0 g/kg of SO into a diet based on maize and soybean-meal tended to attenuate the skatole level in excreta of broiler chickens [8]. Furthermore, SO can also decrease the levels of indole and skatole *in vitro* [14], which may be caused by a decrease in the L-tryptophan degradation rate. SO is cheap and rich in supply. However, there is limited information regarding the interaction between dietary dTry and SO in broiler chickens.

We hypothesized that the interaction between dTry level and SO would attenuate the skatole level by modulating the caecal microflora in broiler chickens. Therefore, the aim of this study was to determine how dietary dTry, SO and their interactions affect growth performance, caecal skatole level and microflora in broiler chickens aged from 14 to 42 days. The basis for trials from 14 days onward was that the levels of indole and skatole in the caecum of 14-day-old chicks were significantly lower than those of 28, 35, and 42-day-old broilers in our previous study [15].

## MATERIALS AND METHODS

### Materials, experimental design, diets, and bird management

All procedures regarding animal handling and treatments were approved by the Institutional Animal Care and Use Committee of Shenyang Agricultural University (approval No. 201708016). The feeding experiment was carried out at Shenyang Agricultural University (Shenyang, China) from April to June 2019.

A total of 400 one-day-old commercial broiler chicks (Arbor Acres plus; Aviagen, Huntsville, AL, USA) were obtained from a commercial hatchery (Shenyang Huamei livestock and poultry Co., Ltd, Shenyang, China). The birds were fed a commercial starter diet to day 14. The diet was formulated with the following specifications: 12.67 MJ/kg apparent metabolic energy, 21.9% crude protein, 0.87% calcium, 0.68% phosphorus, 1.42% lysine, 0.56% methionine, 0.96% threonine, 0.25% tryptophan (calculated), purchased from Shenyang Boeing Feed Co. (Shenyang, China). At the age of 14 days, 360 birds were selected (based on gender and bodyweight) and assigned to 6 treatments with 6 replicated cages and 10 birds (5 female and 5 male) per cage each (1.04 m^2^ in size) (six cages, 60 birds/treatment).

A completely randomised design involving a 3×2 factorial arrangement of treatments was used in this experiment. Factors were dietary dTry levels (0.18%, 0.23%, or 0.28%) and SO supplementation (0 or 3.5 g/kg) (Table 1). SO (powder, off white), was sourced and supplied by Henan Biotechnology Co. (Henan, China), and contained 717.6 g/kg of SO, including 669.8 g/kg of sucrose, 100.8 g/kg of raffinose, and 549.8 g/kg of stachyose, analysed by ultraviolet spectrophotometry using Evolution 220 UV-Vis Spectrophotometer (Thermo Scientific Co., Shanghai, China). Diets were presented in mash form. Diets were formulated based on the Arbor Acres broiler management guide (Beijing Arbor Acres Poultry Breeding Co., Ltd. Beijing, China) and considered the local feed ingredients, and in line with those used in the common starter diet (1 to 14 days). The dTry level in the basal diet was 0.18%, based on the feed standard recommended by the company (Aviagen, USA), and Borges et al [11]. The SO dose chosen is the manufacturer's recommended addition in typical maize-soybean meal diet, and our previous study has proven the dose to be beneficial for broiler chickens [8].

According to the feed formula, first, premixed the feed ingredients except energy and protein feeds, and then mixed with energy and protein feeds. Each experimental diet was fed from 14 to 42 days, which was the duration of this study. The diets were fed *ad libitum*, and all broiler chickens were given free access to water from nipple drinkers. Birds were housed in cages in a temperature- and light-controlled facility with a light schedule of 23 h of light (provided 30–40 lux to 7 days of age, 5–10 lux after 7 days of age) and 1 h of darkness. The house temperature was maintained between 35°C±1°C for the initial 3 days, and it was then decreased by 2°C to 3°C each week until a final temperature of 21°C was obtained. The relative humidity was kept at approximately 70% for the first three days and maintained at 60% to 65% thereafter. Birds were vaccinated with attenuated Newcastle disease vaccines at seven days of age by putting drops in their nose and eyes, and this procedure was repeated when they were 28 days old. Similarly, chicks were vaccinated against Bursal disease at 12 days of age via oral drip, and this treatment was repeated when they were 22 days old.

### Chemical analyses

Diets and feeds were dried at 65°C and ground to pass a 0.45 mm screen before analysis. The dry matter content was determined after drying (105°C oven, to constant weight). The gross calorific values in diets were determined using an IKA C2000 calorimeter (IKA, Staufen, Germany) according to the manufacturer's instructions. Crude protein contents in feeds and diets were assayed by the Kjeldahl method for nitrogen determination [16]. Analysis of amino acids in feeds and diets were measured according to Millet [17] using the Hitachi L8800 amino acid automatic analyzer (Hitachi Limited, Tokyo, Japan) after hydrolysis with 6 mol/L HCl at 110°C for 24 h under reflux. The methionine and cystine contents in feeds and diets were determined after performic acid oxidation before hydrolysis. Tryptophan contents in feeds and diets were determined after hydrolysis at 40°C for 16 h with potassium hydroxide and separation by spectrophotometric method (the wavelength was 590 nm) with the F280 spectrofluorometer (Gangdong Co., Ltd, Tianjin, China). The sucrose, raffinose and stachyose in soybean meal, extruded full-fat soybean, were assayed by the ion chromatography method referred by Bansleben et al [18].

### Growth performance measurement and sample collection

Birds were weighted on a replicate (cage, ten birds) basis on d 14, 28, and 42. Feed intake was determined by replicate biweekly. Mortality was checked daily. Average daily gain (ADG), average daily feed intake (ADFI) and feed:gain ratio (F/G) were calculated for the entire experimental period.

On d 42, one bird from each replicate (three males and three females per treatment) with a body weight close to the replicate cage mean was selected and euthanatized by cervical dislocation. For each bird, both caeca were removed, and then the caecal content was collected by gently squeezing, pooled, and stored as 2 aliquots. One tube was stored at −20°C for analysis of indole, skatole, and volatile fatty acids (VFAs) levels, whereas another was immediately snap-frozen in liquid N_2_ for 15 min and then stored at −80°C for analyses of total microbial community.

### Assessment of indole, skatole, and volatile fatty acids levels

The levels of indole and skatole in the caecal contents were assessed using HPLC equipment (Agilent 1100; Agilent Technologies, Santa Clara, CA, USA). Approximately 1.0 g of caecal content was homogenised with 2 mL of distilled water, mixed, homogenised, and centrifuged at 3,000×*g* for 10 min. One mL supernatant was added to 2 mL of methanol (chromatographic grade). After vortex mixing, it was stored at −20°C for 30 min, and then centrifuged at 15,000×*g* for 30 min (4°C). The supernatant was collected and filtered through a 0.45-μm filter membrane into a brown injection bottle, and the sample injection volume was 20 μL. The chromatographic conditions were as described in Yang et al [19].

The levels of acetate, propionate, and butyrate were measured with a gas chromatograph (7890B; Agilent, USA) equipped with a DIMA-FFAP capillary column (30 m×0.25 mm×0.25 μm) (Agilent Technologies, USA) and a flame ionisation detector. The external standard method was used. Approximately 1.0 g of caecal content was homogenised with 9 mL of distilled water, mixed, homogenised, and centrifuged at 5,000×*g* for 10 min. The supernatant was added to 0.2 mL of 25% metaphosphoric acid, incubated at 4°C for 30 min, and then centrifuged at 15,000×*g* for 10 min. The supernatant liquid was collected and filtered through a 0.22-μm filter membrane into a 1.5-mL centrifuge tube. Gas chromatographic conditions were as described in Julák et al [20], and the injection volume was 1.0 μL. pH was measured using a digital pH meter (PHS-3C, Shanghai, China) after mixing approximately 1.0 g of caecal content with 2 mL of distilled water.

### Total microbial community analysis

#### DNA extraction and polymerase chain reaction amplification

Approximately 0.2 g of caecal content was weighed and put into a 2-mL sterile centrifuge tube. The genomic DNA (gDNA) from the caecal content was extracted using the Stool gDNA Extraction kit (Tiangen Biotechnology Co., Ltd., Beijing, China) following the cetyltrimethylammonium bromide method as previously described [19]. The purity and concentration of DNA were detected by 1.5% agarose gel electrophoresis and a NanoDrop 2000 micro spectrophotometer (Thermo Scientific Inc., Shanghai, China). Bacterial 16S rDNA genes containing the V3-V4 of the variable region were amplified using the primers 341F and 805R (F: CCCTACACGACGCTCTTCCGATCTG, R: GACTGGA GTTCCTTGGCACCCGAGAATTCCA). Polymerase chain reaction (PCR) amplification was performed using PCR Kits (KAPA Biosystems, Inc., Beijing, China). The amplification conditions were as follows: an initial cycle at 95°C for 3 min, and then 30 cycles at 95°C for 30 s, at 50°C for 30 s, at 72°C for 60 s, and finally one cycle at 72°C for 7 min.

The PCR products were mixed in equidensity ratios, and then the mixture of PCR products was purified with the GeneJET Gel extraction kit (Thermo Scientific Inc., China).

#### Library construction and sequencing

The DNA library construction was performed using an NEB Next Ultra DNA library prep kit for Illumina (New England Biolabs, Inc., Beijing, China) following the manufacturer’s recommendations. The library quality was assessed on the Qubit@2.0 Fluorometer (Thermo Scientific Inc., China) and Agilent Bioanalyzer 2100 systems. Finally, the library was sequenced on an Illumina HiSeq platform, and 250 bp paired-end reads were generated.

#### Bioinformatic analysis

After sequencing, the primer connector sequences of the raw sequenced reads were discarded, and the paired reads were merged into one sample read according to the barcode label. Whole sample reads were filtered for quality control and processed to remove nonamplification sequences and chimeric sequences using the Usearch and Uchime software packages (http://drive5.com/uparse), respectively, and assembled to obtain operational sequences using the Uclust algorithm [21]. Sets of sequences with 97% identity were defined as an operational taxonomic unit (OTU). We used the ribosomal database project (RDP) classifier and referred to the GreenGenes database (http://rdp.cme.msu.edu/misc/resources.jsp) for microflora species annotation. The community richness indexes (abundance-based coverage estimator [ACE] and the bias-corrected Chao 1) and the community diversity (Shannon and Simpson indices) were calculated using the Mothur program (http://www.mothur.org/wiki). Based on the multisequence queue, we used the UniFrac algorithm to calculate sample distance. According to the distance between samples, the sample principal coordinate analysis (PCoA) diagram was drawn using the vegan diagram package of the R software, and then the microbial beta diversity was analysed.

### Statistical analyses

The data were analysed using general linear model procedures in SPSS (version 22.0, Chicago, IL, USA) with dietary dTry level (0.18%, 0.23%, or 0.28%) and SO supplementation (0 or 3.5 g/kg) as main factors. The model for all data included the effects of dietary dTry (n = 12), SO (n = 18), and their interaction (n = 6). Percentage values (relative frequency of microflora) were arcsine-transformed before analysis. Results were presented as treatment means, standard errors of the mean (SEM), and p value. Differences among means were tested by Duncan’s multiple range tests, and the statistical significance was set at p<0.05.

## RESULTS

### Growth performance

The broiler chickens were healthy and appeared to be in normal conditions throughout the study. There were no mortalities or culled birds, and no major adverse events occurred during the trial period.

As illustrated in Table 2. The initial and final average body weight of birds were 542 g and 2,105 g, respectively. There was no statistical difference in average bodyweight for any of the cages at the beginning and the finishing of the feeding study. A significantly different dTry level×SO interaction was found for ADG (p<0.05). This interaction demonstrated that birds fed diets containing 0.23% dTry level with SO supplementation had higher ADG than those fed the other diets (p<0.05). This diet also resulted in the lowest F/G (p<0.05). Birds fed diets containing 0.18% dTry level with SO supplementation had higher ADFI than those fed diets containing 0.28% dTry level with SO supplementation (p<0.05). Broiler chickens fed diets containing 0.23% dTry had greater ADG and lower F/G than those fed diets containing 0.18% or 0.28% dTry (p<0.05), regardless of SO addition. SO supplementation increased ADG (p<0.05), and tended to reduced F/G (p = 0.057), regardless of dietary dTry level.

### Levels of skatole, indole, volatile fatty acids, and pH value in caeca

As depicted in Table 3, a significantly different dTry level× SO interaction was found for caecal indole level (p<0.05). This interaction demonstrated that birds fed diet containing 0.18% dTry level had a lower indole level than the other treatments (p<0.05) except for the 0.23%+SO treatment. Birds fed diet containing 0.23% dTry level with SO supplementation had the lowest skatole level (p<0.05), whereas birds fed a diet containing 0.28% dTry level without SO supplementation had the highest level (208.6 ng/g). Birds fed diets containing 0.28% dTry level increased caecal indole and skatole level compared to those fed diets containing 0.18% or 0.23% dTry level (p<0.05), regardless of SO addition. SO supplementation to diets decreased the caecal skatole levels (p<0.05), regardless of dietary dTry level.

Birds fed diet containing 0.18% or 0.28% dTry level without SO supplementation had higher caecal level of acetic acid than those fed the other diets (p<0.05). The levels of propionic acid in the 0.23%, 0.23% + SO, and 0.28% + SO treatments were significantly lower than the other treatments (p<0.05). The levels of butyric acid in the 0.18% + SO, 0.23% + SO, and 0.28% + SO treatments were significantly lower than in the 0.18%, 0.23%, and 0.28% treatments (p<0.05). The acetic, propionic, and butyric acid, and pH values differed among the various dTry levels (p<0.05), regardless of SO addition. SO supplementation to diets decreased the caecal levels of acetic, butyric, and propionic acid and lowered the pH value (p<0.05), regardless of dietary dTry level.

### Species classification of microflora in caecal contents of broiler chickens

#### Bacterial DNA sequence data

As shown in Table 4, the raw sequences, filtered sequences, and OTU numbers were not affected by any of the dietary treatments (p>0.05). Broiler chickens fed diets containing 0.18% dTry level had higher chimera numbers than those fed diets containing 0.28% dTry level (p<0.05), regardless of SO addition.

#### Microbial species annotation

**Figure 1:**
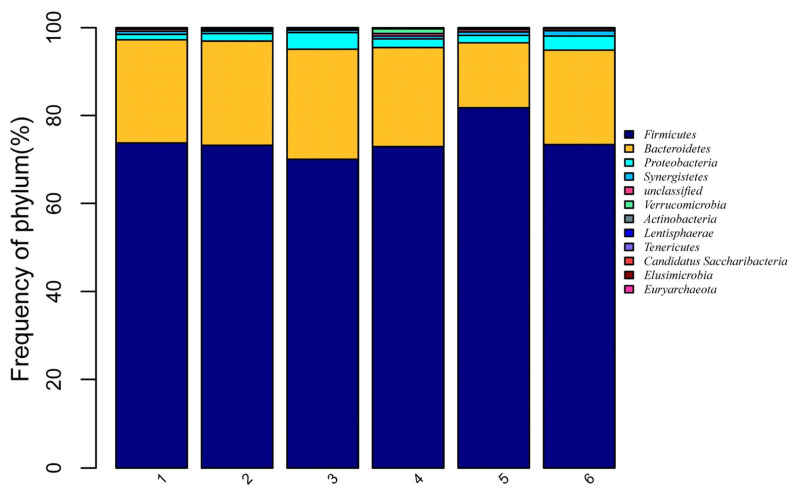
Effects of dietary digestible tryptophan (dTry) and soy oligossacharide (SO) supplementation on caecal microbial relative abundances in broiler chickens (at phylum level). The distribution barplot was drawn by using the barplot package of the R software. 1, 2, 3, 4, 5, 6 represent the 0.18%, 0.18%+SO, 0.23%, 0.23%+SO, 0.28%, 0.28%+SO treatments, respectively. 0.18%, 0.23%, and 0.28% refer to dTry level.

A total of 12 phyla and 40 genera were obtained from the 36 broiler caecal samples. There were eight phyla with relative frequency greater than 0.1% (Figure 1). These were the following: *Firmicutes*, *Bacteroidetes*, *Proteobacteria*, *Synergistetes*, Unclassified, *Verrucomicrobia*, *Actinobacteria*, and Others. *Firmicutes* was the dominant phylum, accounting for 74.3% of the overall bacterial community, followed by *Bacteroidetes* at 21.7%. However, no significantly difference was observed among the treatments (p>0.05).

Tables 5-i), and 5-ii) depict the structure of the bacterial community of all treatments in the broiler caecal samples at the genus level. The sequences were more abundant for unclassified genera, with an average relative abundance of >39.9% across all treatments. The major classified genera with relative frequencies greater than 1.0% across all treatments included *Bacteroides* at 10.6%, *Alistipes* at 7.3%, *Sporobacter* at 5.0%, *Lactobacillus* at 3.3%, *Ruminococcus* at 3.3%, *Faecalibacterium* at 3.2%, *Clostridium IV* at 2.8%, *Pseudoflavonifractor* at 2.0%, *Clostridium XlVa* at 2.0%, *Butyricicoccus* at 2.0%, *Subdoligranulum* at 1.9%, *Parabacteroides* at 1.6%, *Intestinimonas* at 1.5%, and *Clostridium XlVb* at 1.2%. Birds fed diet containing 0.23% dTry level with SO supplementation had higher *Clostridium IV* relative frequency (3.6%) than those fed diet containing dTry level of 18% or 0.23% without SO supplementation, and 0.28% dTry level with SO supplementation (p<0.05). SO supplementation to diets increased the relative frequency of *Clostridium IV* (p<0.05), regardless of dietary dTry level.

Caecal microbial alpha diversity analysis: As depicted in Table 6, the sequencing Goods coverage of the samples was above 0.994, thus reflecting the real situation of the microflora in the samples. There was no difference in terms of the ACE, Chao1, and Simpson indices or Goods coverage among the treatments (p>0.05). A significant different dTry level× SO interaction was found for the microbial Shannon index in the caeca of broiler chickens (p<0.05). This interaction demonstrated that birds fed diet containing 0.28% dTry level without SO supplementation had the highest Shannon index (4.91), and it was higher than those fed diet containing 0.23% dTry level without SO supplementation, and 0.28% dTry level with SO supplementation (p<0.05). However, it did not differ significantly from the other treatments (p>0.05).

#### Caecal microbial beta diversity (similarity) analysis

**Figure 2:**
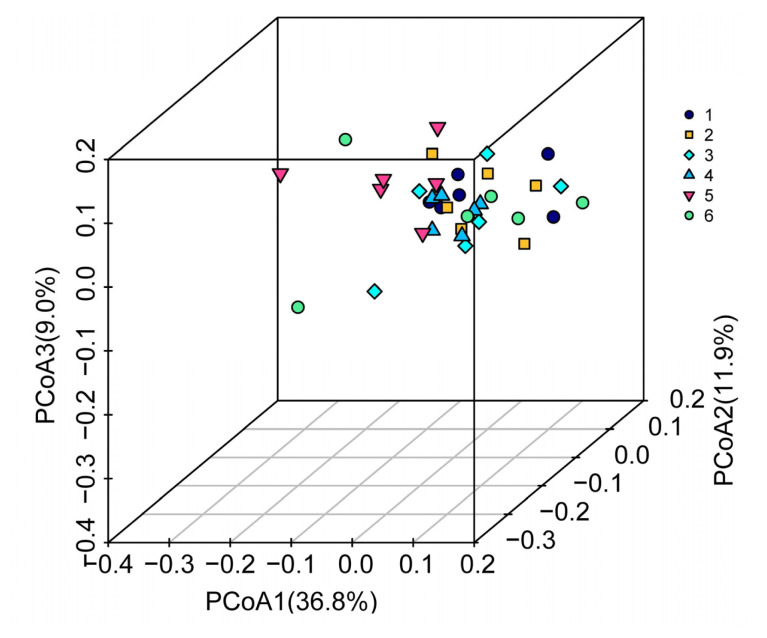
Multi-sample principal co-ordinate analysis (PCoA) analyses based on weighted_unifrac matrix. The three-dimensional plot was drawn by using the vegan diagram package of the R software. Dots of different colours represent samples in different groups; the higher the similarity between samples, the more aggregation in the graph. Otherwise, the lower the similarity between samples, the greater the spatial distance. 1, 2, 3, 4, 5, 6 represent the 0.18%, 0.18%+SO, 0.23%, 0.23%+SO, 0.28%, 0.28%+SO treatments, respectively. 0.18%, 0.23%, and 0.28% refer to dTry level.

To determine if individuals of a treatment clustered together when considering the entire microflora, a three-dimensional plot of samples based on PCoA was constructed (Figure 2). The percentages of variation explained by PCoA 1, PCoA 2, and PCoA 3 were 36.8%, 11.9%, and 9.0%, respectively. Dots of different colours represent samples in different treatments. The higher the similarity between samples, the more aggregation in the graph; otherwise, the lower the similarity between samples, the greater the spatial distance. The highest density was found among the samples that originated from the birds fed diet containing 0.23% dTry level with SO supplementation, whereas the samples from the 0.28% dTry level with SO supplementation exhibited the lowest.

## DISCUSSION

### Interactive effects of dietary digestible tryptophan and soy oligosaccharide on growth performance of broiler chickens

Among essential amino acids, tryptophan stands out for participating in protein synthesis and being a precursor of serotonin, which is related to the stimulation of feed intake [22]. Many studies have shown that a deficiency or excess of dietary tryptophan can lead to the decrease of ADG in broiler chickens [11]. Wang et al [10] reported the highest ADG from 21 to 42 d when male broilers were fed diets containing 0.15% apparent ileal dTry. Opoola et al [23] indicated that broilers reared in the tropical region may require 0.18% dietary tryptophan for 33 to 56 days based on growth performance. Based on their growth performance outcome, it is suggested that the dTry level of 0.23% resulted in the highest ADG and the lowest F/G, regardless of SO addition. This value is higher than the recommendation of Arbor Acres’s genetic line 0.18% for the period of grower (11 to 24 d) and finisher (25 d to market). The dynamic genetic improvement of strains of broiler chickens, raising method (cages vs floor pens), size of the study (i.e., number of birds) etc., may be the reason for their differences. Thus, owing to nutritional requirement of broiler chickens vary constantly, continuous research is needed.

SO has diverse effects on promoting animal growth, digestion, and metabolism. Our previous studies have shown that for broiler chickens 21 to 42 days of age, the optimum addition of SO is approximately 3.5 g/kg [8]. In this study, diets supplemented with 3.5 g/kg SO had significant effects on ADG of broiler chickens 14 to 42 days of age, regardless of dietary dTry level. This result was consistent with those of other authors [8,19], and there was a significant interaction between dTry and SO on ADG. The growth rates of broiler chickens were highest in the diet containing 0.23% dTry level with SO supplementation.

### Interactive effects of dietary digestible tryptophan and soy oligosaccharide on levels of skatole, volatile fatty acids and pH value in caecal contents of broiler chickens

Indole and skatole result from a multistep degradation of L-tryptophan via microbial activity, and they contribute to malodourous chicken excreta [24]. Tryptophan in the diet and endogenous tryptophan in the intestinal tract are directly related to the production of indole and skatole. Skatole in the pig caecum and colon is mainly produced by endogenous tryptophan in intestinal epithelial cells but is undetected in the stomach and small intestine [25]. Cho et al [26] reported that when dietary protein was reduced from 18% to 12%, the skatole levels in pig faeces were reduced by 40%. Similarly, Khattak and Helmbrecht [4] demonstrated that levels of indole and skatole in the caeca of laying hens were significantly reduced as dietary dTry levels decreased from 0.31% to 0.10%.

The existing database regarding the tryptophan requirement for broilers lacks consistency [11]. In this study, broilers fed diets containing 0.28% dTry had higher caecal levels of indole and skatole than those fed diets containing 0.18% and 0.23% dTry. This result was consistent with that of Khattak and Helmbrecht [4]. Therefore, it can be speculated that an excess of tryptophan would pass to the caecum, becoming a potential target for nitrogen-fermenting bacteria, and there it would be converted into potentially harmful indole and skatole [12].

It has been shown that the inclusion of 3.5 to 5.0 g/kg of SO to a diet based on maize- soybean meal tended to decrease the skatole level in excreta of broiler chickens [8]. Similarly, the present study indicated that the supplementation of SO significantly reduced the caecal skatole levels. As a microbial energy substance, SO can enter the hindgut of broilers, and be preferentially used in fermentation, thus reducing the fermentation of tryptophan by bacteria [12] and thereby reducing the levels of skatole and indole.

The VFAs are metabolites of gut microflora. The proportion and quantity of VFAs are different in different animals. Macfarlane et al [27] reported that the substrates used in bacterial fermentation would affect the production of VFAs. Liu et al [28] showed that broilers in the stachyose -fed treatment had higher caecal acetate, propionate and the butyrate level compared with the control group. Zhou et al [29] found that the *in vitro* addition of 2% SO into the fermentation broth of pig caecal content increased the levels of acetic acid, propionic acid, and butyric acid. Lan et al [30] also reported that dietary SO increased the levels of acetic, propionic, and butyric acid in the *in vitro* fermentation of broiler caecal content. Similarly, Liu et al [14] showed that supplementation of SO increased the acetic acid level *in vitro*. Zhu et al [7] indicated that dietary SO supplementation increased caecal VFAs and decreased lactic acid levels in broiler chickens.

Results from this study showed that dietary dTry level had a significant impact on caecal acetic, propionic acid, and pH values of broiler chickens. The levels of acetic, propionic acid were higher in 0.18% and 0.28% dTry treatments but were lower in 0.23% dTry level. Zhao et al [31] reported that moderate protein levels can lead to an increase in *Lactobacillus* and reduction of *Coliforms* and *Staphylococci* in the intestine. When the level of dietary protein is greater, there are increases in the populations of pathogens, such as *Coliforms*, *Streptococcus* and *Bacillus* [32]. However, when the concentration of protein in the diet is too low to meet the basic requirement for host, it can increase the abundance of potentially pathogens and decrease the population of prebiotics [31], then affect microbial metabolism. In addition, the amino acid balance is a major factor affecting the protein digestibility in body. This suggests that 0.23% dTry level may lead to an increase in the population of prebiotics.

The supplementation of SO in diets decreased VFAs levels in the caeca of broiler chickens. The results are inconsistent with the above literature reports [7,14]. This difference may suggest the influence of SO on the number and activity of acid-producing bacteria in the cecum via changing the proportion of dominant bacteria such as *Bifidobacterium* and *Lactobacillus* and reducing the levels of other VFAs. In addition, in this study the dTry and SO had significantly interaction effects on caecal propionic and butyric acid levels. Therefore, we speculate that dietary dTry plays a leading role in this interaction, whereas SO plays a secondary role.

It is worth noting that the level of VFAs in the caeca does not necessarily reflect the rate of its production by bacteria [33]. Approximately 95% to 99% of VFAs produced in the hindgut are absorbed [34]. In addition, caecal contents flow is dynamic, and reverse peristalsis may result in variability in local levels of different VFAs [35]. Thus, the results suggest the mechanisms by which these effects are brought about are poorly comprehended.

### Interactive effects of dietary digestible tryptophan and soy oligosaccharide on microflora in caecal contents of broiler chickens

Animal intestinal microflora play an important role in the degradation of undigested proteins and carbohydrates. The intestinal microbiota communities are influenced by components of dietary protein (amino acid), specifically the protein sources and levels in diet [31]. Zhou et al [36] suggested that there was a significant correlation between the skatole level and certain bacterial genera such as *Clostridium* and *Oscillibacter* in pig faeces, with the increase of tryptophan levels, the microbial alpha diversity in the pig colon increased. Liu et al. [14] suggested that the supplementation of SO promotes the growth of uncultured *Firmicutes*, *Pullorum*, and *Bacteroides* in the caecal microbiota fermentation broth of broilers. In our study, the supplementation of SO in diets increased the relative frequency of *Clostridium IV*. Research suggests that *Clostridium IV* and *Bacteroides* play important roles in nutrient absorption, VFA production, and maturation and maintenance of intestinal epithelial cells [37]. The abundance of *Clostridium IV* showed negative indole correlations and positive skatole correlations in the faeces of pigs [36]. Whether *Clostridium IV* was related to the caecal skatole production in broilers needs further study.

Shannon and Simpson indexes are often used to reflect microbial alpha diversity. The higher the Shannon value, the higher the community diversity. In contrast, the higher the Simpson index, the lower the community diversity. In our study, the highest Shannon index was observed in the 0.28% dTry level group. However, the similarity of microflora in the 0.23% dTry level with supplementation SO treatment displayed the best microbial stability (beta diversity, Figure 2). The reason for this result remains unclear.

Although both indole and skatole are the end products of tryptophan fermentation, they may be produced by different bacteria. Research suggests that in the distal intestine, saccharolytic fermentation is preferred, and putrefaction accelerates only when utilisable carbohydrates are depleted [12]. When carbohydrates in the distal intestine become depleted, putrefaction becomes the dominant type of fermentation [38]. This largely accounts for the health effects of prebiotics such as oligosaccharides [7,8,19], and soluble non-starch polysaccharides [9]. Therefore, we speculated that the effect of SO on skatole reduction in the caecum may be related to the improvement of microflora structure through suitable levels of dTry supplements with the SO supplementation diet.

## CONCLUSION

Thus, we concluded that there were significant interactions between dTry level and SO on ADG, caecal levels of indole, propionic acid, and butyric acid, and microbial Shannon index of broiler chickens. Broilers fed diets containing 0.23% dTry with 3.5 g/kg of SO supplementation displayed an increased growth performance and a decreased caecal skatole levels.

## Figures and Tables

**Table 1:** Ingredients and chemical composition of the experimental diets for broiler chickens aged from 14 to 42 days (%, as-fed)

Ingredients	Dietary treatments						

	dTry level	0.18%		0.23%		0.28%	
		
	SO inclusion	No	Yes	No	Yes	No	Yes
Maize		52.27	52.27	52.27	52.27	52.27	52.60
Soybean meal		7.52	7.24	7.54	7.19	7.50	6.81
Rice chaff		7.00	7.00	7.00	7.00	7.00	7.00
DDGS		5.00	5.00	5.00	5.00	5.00	5.00
Fish meal		4.50	4.50	4.50	4.50	4.50	4.50
Extruded full-fat soybean		18.77	18.77	18.77	18.77	18.77	18.77
Soy oil		2.07	2.00	2.00	2.00	2.00	2.00
Limestone (CaCO_3_)		0.73	0.73	0.73	0.73	0.73	0.73
CaHPO_4_		1.00	1.00	1.00	1.00	1.00	1.00
Vitamin premix^1)^		0.05	0.05	0.05	0.05	0.05	0.05
Mineral element premix^2)^		0.10	0.10	0.10	0.10	0.10	0.10
Salt (NaCl)		0.15	0.15	0.15	0.15	0.15	0.15
Choline chloride		0.10	0.10	0.10	0.10	0.10	0.10
L-Lysine HCl, 78%		0.26	0.26	0.26	0.26	0.26	0.26
DL-Methionine, 99%		0.28	0.28	0.28	0.28	0.28	0.28
L-Threonine, 98%		0.20	0.20	0.20	0.20	0.20	0.20
L-Tryptophan, 98%		0	0	0.04	0.04	0.09	0.09
Antioxidant		0.01	0.01	0.01	0.01	0.01	0.01
Soy oligosaccharide		0	0.35	0	0.35	0	0.35
Total		100.00	100.00	100.00	100.00	100.00	100.00
Calculated (analyzed) nutritional levels^3)^							
Gross energy (MJ/kg)		(18.54)	(19.28)	(18.72)	(18.57)	(18.69)	(19.31)
Metabolisable energy (MJ/kg)		13.40	13.40	13.39	13.39	13.38	13.38
Dry matter		89.20 (88.74)	89.20 (88.93)	89.20 (89.02)	89.20 (88.69)	89.20 (88.90)	89.20 (89.04)
Crude protein		17.67 (17.58)	17.67 (17.58)	17.71 (17.75)	17.71 (17.75)	17.75 (18.10)	17.75 (18.10)
Ca		1.02 (1.11)	1.02 (1.15)	1.02 (1.11)	1.02 (1.11)	1.02 (1.11)	1.02 (1.11)
Total P		0.75 (0.76)	0.75 (0.73)	0.75 (0.70)	0.75 (0.69)	0.75 (0.74)	0.75 (0.71)
Available P		0.37	0.37	0.37	0.37	0.37	0.37
Total lysine		1.29 (1.26)	1.29 (1.27)	1.29 (1.26)	1.29 (1.27)	1.29 (1.25)	1.29 (1.26)
Total methionine		0.61 (0.59)	0.61 (0.58)	0.61 (0.61)	0.61 (0.58)	0.61 (0.60)	0.61 (0.59)
Total threonine		0.93 (0.96)	0.93 (0.95)	0.93 (0.97)	0.93 (0.95)	0.93 (0.96)	0.93 (0.94)
Total tryptophan		0.21 (0.20)	0.21 (0.21)	0.26 (0.24)	0.26 (0.25)	0.31 (0.29)	0.31 (0.29)
SID lysine		1.14	1.14	1.14	1.14	1.14	1.14
SID methionine		0.57	0.57	0.57	0.57	0.57	0.57
SID threonine		0.80	0.80	0.80	0.80	0.80	0.80
SID tryptophan		0.18	0.18	0.23	0.23	0.28	0.28
Soy oligosaccharide^4)^		1.16	1.40	1.16	1.40	1.16	1.38

DDGS, dried distillers grains with solubles; dTry, digestible tryptophan; SO, soy oligosaccharide (No, 0 g/kg or Yes, 3.5 g/kg); SID, standardized ileal digestible.

1) Provided per kilogram of diet: vitamin A 10,000 IU; vitamin D_3_ 1,000 IU; vitamin E 25 IU; vitamin K 0.5 mg; vitamin B_1_ 2 mg; vitamin B_2_ 8.0 mg; vitamin B_6_ 4.5 mg; vitamin B_12_ 0.01 mg; biotin 0.25 mg; folic acid 0.50 mg; niacin 40 mg; pantothenic acid 10 mg; niacin 40 mg.

2) Provided per kilogram of diet: Cu (CuSO_4_·5H_2_O) 25 mg; I (KI) 1.0 mg; Fe (FeSO_4_·7H_2_O) 100 mg; Mn (MnSO_4_·H_2_O) 120 mg; Se (NaSeO_3_) 0.15 mg; Zn (ZnO) 80 mg.

3) Nutrient levels are calculated values, and the measured values are in parentheses.

4) Soy oligosaccharide contents (sum of sucrose, raffinose and stachyose) are calculated values according to the analysed values of the soybean meal, extruded full-fat soybean, and soy oligosaccharide product.

**Table 2:** Effects of dietary digestible tryptophan (dTry) and soy oligossacharide (SO) supplementation on growth performance of broilers aged from 14 to 42 days

Treatments		Initial weight (g)	Final weight (g)	Average daily feed intake (g/d)	Average daily gain (g/d)	Feed/gain (g/g)
dTry level	SO inclusion					
0.18%	No	545.0	2,065	105.1^ab^	54.3^d^	1.95^a^
	Yes	539.2	2,110	107.4^a^	57.8^c^	1.86^a^
0.23%	No	539.2	2,168	104.7^ab^	63.2^b^	1.66^b^
	Yes	540.0	2,088	104.9^ab^	67.5^a^	1.56^b^
0.28%	No	542.5	2,115	104.7^ab^	56.2^cd^	1.87^a^
	Yes	543.3	2,086	102.4^b^	55.1^cd^	1.86^a^
SEM		2.60	32.9	1.03	0.94	0.04
Main effects						
dTry level	0.18%	542.1	2,088	106.3^a^	56.0^b^	1.91^a^
	0.23%	539.2	2,128	104.8^ab^	65.3^a^	1.61^b^
	0.28%	543.3	2,100	103.6^b^	55.6^b^	1.86^a^
SEM		1.84	23.3	0.73	0.67	0.028
SO	No	542.2	2,116	104.9	57.9^b^	1.83^x^
	Yes	540.8	2,095	104.9	60.1^a^	1.76^y^
SEM		1.50	19.0	0.59	0.54	0.02
p-value						
dTry level×SO		0.504	0.182	0.093	0.016	0.434
dTry level		0.420	0.464	0.043	<0.001	<0.001
SO		0.517	0.438	0.964	0.007	0.057

dTry, digestible tryptophan; SO, soy oligosaccharide (No, 0 g/kg or Yes, 3.5 g/kg); SEM, standard error of the mean.

a–d Data within a column within treatment without a common superscript letter mean significant difference (p<0.05).

x–y Without a common superscript letter mean significant difference trend (0.05<p<0.1).

**Table 3:** Effects of dietary digestible tryptophan (dTry) and soy oligossacharide (SO) supplementation on levels of skatole, indole, volatile fatty acids, and pH value in caecal contents of broiler chickens

Treatments		Indole (ng/g)	Skatole (ng/g)	pH value	Acetic acid (mg/g)	Propionic acid (mg/g)	Butyric acid (mg/g)
dTry level	SO inclusion						
0.18%	No	137.2^b^	137.4^b^	6.24^b^	3.83^a^	0.61^a^	1.61^a^
	Yes	215.1^a^	116.7^b^	6.16^b^	2.53^b^	0.51^a^	0.65^d^
0.23%	No	212.4^a^	136.3^b^	6.18^b^	2.91^b^	0.22^b^	1.01^c^
	Yes	146.4^b^	103.2^c^	6.03^c^	2.17^b^	0.27^b^	0.67^d^
0.28%	No	244.1^a^	208.6^a^	6.38^a^	3.98^a^	0.64^a^	1.3^b^
	Yes	244.7^a^	184.4^a^	6.33^a^	2.66^b^	0.27^b^	0.78^cd^
SEM		27.51	31.25	0.056	0.26	0.046	0.13
Main effects							
dTry level	0.18%	176.1^b^	127.0^b^	6.20^b^	3.18^a^	0.56^a^	1.13^a^
	0.23%	179.4^b^	119.8^b^	6.12^c^	2.55^b^	0.24^b^	0.84^b^
	0.28%	244.4^a^	196.5^a^	6.34^a^	3.32^a^	0.45^a^	1.04^ab^
SEM		19.75	22.43	0.040	0.19	0.033	0.092
SO	No	197.9	160.8^a^	6.26^a^	3.57^a^	0.49^a^	1.31^a^
	Yes	202.1	134.8^b^	6.17^b^	2.45^b^	0.35^b^	0.70^b^
SEM		16.07	18.25	0.033	0.15	0.027	0.075
p-value							
dTry level×SO		0.017	0.810	0.220	0.380	0.011	0.016
dTry level		0.005	<0.001	<0.001	0.007	<0.001	0.026
SO		0.630	0.004	0.002	<0.001	0.015	<0.001

dTry, digestible tryptophan; SO, soy oligosaccharide (No, 0 g/kg or Yes, 3.5 g/kg); SEM, standard error of the mean.

a–d Data within a column within treatment without a common superscript letter mean significant difference (p<0.05).

**Table 4:** Effects of dietary digestible tryptophan and soy oligossacharide supplementation on microflora DNA sequence data in caecal contents of broiler chickens

Treatments		Raw reads number	Chimeras number	Filtered sequence number	OTUs number
dTry level	SO inclusion				
0.18%	No	91,239	11,552	79,685	1,184
	Yes	92,869	7,724	85,143	1,199
0.23%	No	88,605	6,583	82,019	1,127
	Yes	80,140	6,454	73,685	1,124
0.28%	No	83,338	3,969	79,367	1,118
	Yes	84,564	6,460	78,102	1,063
SEM		5,373.6	1,730.0	4,886.4	57.5
Main effects					
dTry level	0.18%	92,054	9637^a^	82,414	1,192
	0.23%	84,372	6,518^ab^	77,852	1,125
	0.28%	83,951	5,214^b^	78,734	1,091
SEM		3,799.7	1,223.3	3,455.2	40.6
SO	No	87,727	7,368	80,356	1,143
	Yes	85,857	6,879	78,976	1,129
SEM		3,102.5	998.8	2,821.2	33.2
p-value					
dTry level×SO		0.574	0.203	0.381	0.821
dTry level		0.253	0.045	0.617	0.218
SO		0.673	0.732	0.732	0.762

OTUs, operational taxonomic units; dTry, digestible tryptophan; SO, soy oligosaccharide (No, 0 g/kg or Yes, 3.5 g/kg); SEM, standard error of the mean.

a,b Data within a column within treatment without a common superscript letter mean significant difference (p<0.05).

**Table 5 i):** Effects of dietary digestible tryptophan and soy oligossacharide supplementation on caecal microbial relative abundances in broiler chickens (at genus level)

Items	Treatments							SEM

	dTry level	0.18%		0.23%		0.28%		
		
	SO	No	Yes	No	Yes	No	Yes	
Unclassified		39.85	38.68	34.45	37.08	50.38	38.99	4.321
*Bacteroides*		10.98	10.85	13.89	11.85	3.72	12.37	2.587
*Alistipes*		8.21	7.21	7.95	6.25	8.05	6.11	1.283
*Sporobacter*		4.99	5.27	7.98	4.21	3.65	3.99	1.794
*Lactobacillus*		2.26	3.01	5.04	3.02	3.13	3.08	0.977
*Ruminococcus*		2.48	2.82	2.86	4.37	4.02	3.31	0.893
*Faecalibacterium*		5.24	3.79	1.73	1.88	2.08	4.60	1.359
*Clostridium IV*		2.37^b^	3.25^ab^	2.07^b^	3.64^a^	2.14^b^	3.14^ab^	0.375
*Pseudoflavonifractor*		2.56	2.16	1.10	2.40	2.67	1.35	0.703
*Clostridium XlVa*		1.46	1.48	2.60	2.01	2.12	2.14	0.420
*Butyricicoccus*		1.68	2.12	2.03	2.71	1.71	1.62	0.558
*Subdoligranulum*		2.59	2.34	1.98	1.44	1.32	1.41	0.526
*Parabacteroides*		1.16	2.64	1.12	2.81	1.16	0.84	0.759
*Intestinimonas*		1.55	1.53	1.16	1.73	1.61	1.65	0.305
*Clostridium XlVb*		1.26	1.71	1.03	1.22	0.91	1.17	0.291

dTry, digestible tryptophan; SO, soy oligosaccharide (No, 0 g/kg or Yes, 3.5 g/kg); SEM, standard error of the mean.

a,b Data within a row within treatment without a common superscript letter mean significant difference (p<0.05).

**Table 5. ii):** Effects of dietary digestible tryptophan and soy oligossacharide supplementation on caecal microbial relative abundances in broiler chickens (at genus level)

Main effects	dTry level			SEM	SO		SEM	p-values		
		
	0.18%	0.23%	0.28%		No	Yes		dTry level	SO	dTry level×SO
Unclassified	39.26	35.77	44.69	3.055	41.56	38.25	2.495	0.132	0.356	0.261
*Bacteroides*	10.92	12.87	8.04	1.829	9.53	11.69	1.494	0.189	0.316	0.105
*Alistipes*	7.71	7.10	7.08	0.907	8.07	6.52	0.741	0.858	0.151	0.930
*Sporobacter*	5.13	6.09	3.82	1.268	5.54	4.49	1.036	0.455	0.482	0.432
*Lactobacillus*	2.64	4.03	3.11	0.691	3.48	3.04	0.564	0.362	0.585	0.359
*Ruminococcus*	2.65	3.62	3.67	0.631	3.12	3.50	0.56	0.448	0.610	0.472
*Faecalibacterium*	4.52	1.80	3.34	0.961	3.02	3.42	0.785	0.153	0.716	0.355
*Clostridium IV*	2.81	2.85	2.64	0.265	2.19^b^	3.34^a^	0.217	0.838	0.001	0.625
*Pseudoflavonifractor*	2.36	1.75	2.01	0.497	2.11	1.97	0.406	0.691	0.806	0.184
*Clostridium XlVa*	1.47	2.30	2.13	0.297	2.06	1.88	0.243	0.130	0.598	0.714
*Butyricicoccus*	1.90	2.37	1.66	0.395	1.80	2.15	0.322	0.445	0.456	0.779
*Subdoligranulum*	2.46	1.71	1.37	0.372	1.96	1.73	0.304	0.121	0.591	0.840
*Parabacteroides*	1.90	1.96	1.00	0.537	1.15	2.10	0.438	0.377	0.137	0.359
*Intestinimonas*	1.54	1.44	1.63	0.216	1.44	1.64	0.176	0.830	0.438	0.569
*Clostridium XlVb*	1.48	1.13	1.04	0.206	1.07	1.37	0.168	0.289	0.219	0.894

dTry, digestible tryptophan; SO, soy oligosaccharide (No, 0 g/kg or Yes, 3.5 g/kg); SEM, standard error of the mean.

a,b Data within a row within treatment without a common superscript letter mean significant difference (p<0.05).

**Table 6:** Effects of dietary digestible tryptophan (dTry) and soy oligossacharide (SO) supplementation on microflora richness and diversity index in caecal contents of broiler chickens

Treatments		Richness (ACE index)	Richness (Chao1 index)	Diversity (Shannon index)	Diversity (Simpson index)	Diversity (Goods coverage)
dTry level	SO inclusion					
0.18%	No	2,489.7	2,003.4	4.68^ab^	0.028	0.994
	Yes	2,745.9	2,065.6	4.65^ab^	0.028	0.994
0.23%	No	2,345.0	1,857.8	4.53^b^	0.033	0.994
	Yes	2,340.0	1,883.8	4.78^a^	0.024	0.994
0.28%	No	2,254.4	1,831.9	4.91^a^	0.020	0.995
	Yes	2,137.5	1,720.6	4.57^b^	0.029	0.995
SEM		192.1	119.8	0.098	0.0048	0.00046
Main effects						
dTry level	0.18%	2,617.8	2,034.5	4.67	0.028	0.994
	0.23%	2,342.5	1,870.8	4.65	0.028	0.994
	0.28%	2,196.0	1,776.3	4.74	0.025	0.995
SEM		135.8	84.7	0.069	0.0034	0.00032
SO	No	2,363.0	1,897.7	4.71	0.027	0.994
	Yes	2,407.8	1,890.0	4.66	0.027	0.994
SEM		110.9	69.15	0.057	0.0028	0.00026
p-value						
dTry level×SO		0.614	0.749	0.024	0.183	0.936
dTry level		0.100	0.110	0.649	0.678	0.145
SO		0.777	0.938	0.587	0.911	1.000

dTry, digestible tryptophan; SO, soy oligosaccharide (No, 0 g/kg or Yes, 3.5 g/kg); SEM, standard error of the mean.

a,b Data within a column within treatment without a common superscript letter mean significant difference (p<0.05).
